# Serotonin and dopamine transporter availability in social anxiety disorder after combined treatment with escitalopram and cognitive-behavioral therapy

**DOI:** 10.1038/s41398-022-02187-3

**Published:** 2022-10-07

**Authors:** Olof Hjorth, Andreas Frick, Malin Gingnell, Jonas Engman, Johannes Björkstrand, Vanda Faria, Iman Alaie, Per Carlbring, Gerhard Andersson, My Jonasson, Mark Lubberink, Gunnar Antoni, Margareta Reis, Kurt Wahlstedt, Mats Fredrikson, Tomas Furmark

**Affiliations:** 1 Department of Psychology, Uppsala University, Uppsala, Sweden; 2 The Beijer Laboratory, Department of Medical Sciences, Psychiatry, Uppsala University, Uppsala, Sweden; 3 Department of Medical Sciences, Psychiatry, Uppsala University, Uppsala, Sweden; 4 Department of Psychology, Lund University, Lund, Sweden; 5 Center for Pain and the Brain, Department of Anesthesiology Perioperative and Pain Medicine, Boston Children’s Hospital, Harvard Medical School, Boston, MA USA; 6 Smell & Taste Clinic, Department of Otorhinolaryngology, TU Dresden, Dresden, Germany; 7 Department of Medical Sciences, Child and Adolescent Psychiatry, Uppsala University, Uppsala, Sweden; 8 Department of Psychology, Stockholm University, Stockholm, Sweden; 9 Department of Clinical Neuroscience, Karolinska Institutet, Stockholm, Sweden; 10 Department of Behavioural Sciences and Learning, Department of Biomedical and Clinical Sciences, Linköping University, Linköping, Sweden; 11 Department of Surgical Sciences, Radiology, Uppsala University, Uppsala, Sweden; 12 Department of Medicinal Chemistry, Uppsala University, Uppsala, Sweden; 13 Department of Biomedical and Clinical Sciences, Linköping University, Linköping, Sweden

**Keywords:** Predictive markers, Human behaviour

## Abstract

Selective serotonin reuptake inhibitors (SSRIs) and internet-based cognitive behavioral therapy (ICBT) are recommended treatments of social anxiety disorder (SAD), and often combined, but their effects on monoaminergic signaling are not well understood. In this multi-tracer positron emission tomography (PET) study, 24 patients with SAD were randomized to treatment with escitalopram+ICBT or placebo+ICBT under double-blind conditions. Before and after 9 weeks of treatment, patients were examined with positron emission tomography and the radioligands [^11^C]DASB and [^11^C]PE2I, probing the serotonin (SERT) and dopamine (DAT) transporter proteins respectively. Both treatment combinations resulted in significant improvement as measured by the Liebowitz Social Anxiety Scale (LSAS). At baseline, SERT-DAT co-expression was high and, in the putamen and thalamus, co-expression showed positive associations with symptom severity. SERT-DAT co-expression was also predictive of treatment success, but predictor-outcome associations differed in direction between the treatments. After treatment, average SERT occupancy in the SSRI + ICBT group was >80%, with positive associations between symptom improvement and occupancy in the nucleus accumbens, putamen and anterior cingulate cortex. Following placebo+ICBT, SERT binding increased in the raphe nuclei. DAT binding increased in both groups in limbic and striatal areas, but relations with symptom improvement differed, being negative for SSRI + ICBT and positive for placebo + ICBT. Thus, serotonin-dopamine transporter co-expression exerts influence on symptom severity and remission rate in the treatment of social anxiety disorder. However, the monoamine transporters are modulated in dissimilar ways when cognitive-behavioral treatment is given concomitantly with either SSRI-medication or pill placebo.

## Introduction

Social anxiety disorder (SAD) is a debilitating and chronic psychiatric disorder associated with severe suffering for the individual, negative impact on working-life and relationships [1] and high barriers to seek treatment [2]. Existing treatments, predominantly cognitive behavioral therapy (CBT) and selective serotonin reuptake inhibitors (SSRIs), are often successful [3] and combining the two may further enhance clinical efficacy [4]. Neuroimaging studies support that CBT, SSRIs, as well as combined treatment attenuate amygdala activity and connectivity during emotional conditions in SAD [4–10]. Despite these advances, response rates for first line treatments are only 50–65%, indicating that many patients do not achieve remission [11]. A better understanding of the biological mechanisms underlying treatment efficacy in SAD is therefore needed.

Serotonin has been implicated as a key neurotransmitter in the neurobiology of anxiety [12]. Positron emission tomography (PET) studies of serotonin synthesis capacity, serotonin 1A-receptor and transporter availability have suggested increased pre-synaptic serotonin activity in SAD patients [13–15] and the beneficial effects of SSRIs [3] further point to serotonergic involvement. The primary action of SSRIs is to block the serotonin transporter (SERT) protein that facilitates transmembrane reuptake of serotonin into the pre-synaptic cell [16] and 76–85% occupancy has been suggested to exert efficient symptom relief [17, 18]. Adequate SSRI occupancy of the SERT has been verified in SAD [19, 20]. However, it is not clear if occupancy rate is linearly related to clinical SSRI responses. In a previous PET study, we failed to demonstrate such a relationship in SAD [20], consistent with several studies of major depression [18, 21–23], although it has been observed for certain subpopulations of depression [24, 25]. Despite the well-characterized blockade of SERT by SSRIs, the anxiolytic mechanism of action is still debated [26–28]. A number of downstream effects have been proposed to mediate the clinical effect, for example interactions with dopamine signaling [29–31].

An emerging body of evidence points to the importance of dopamine in SAD [32–37]. While single photon emission computed tomography (SPECT) studies of the dopamine transporter (DAT) have yielded mixed findings [35, 38–40], we recently demonstrated, by use of PET, that SAD symptom severity was associated with increased striatal DAT binding and that DAT-SERT co-expression was higher in SAD patients relative to healthy controls [15]. There is considerable interaction between the serotonin and dopamine systems [41–43], for example, DATs may contribute to serotonin reuptake [44–47]. Molecular imaging studies have demonstrated changes in DAT availability with SSRI treatment [48–52] suggesting that SSRIs could exert secondary effects on the DAT. We recently showed that striatal DAT binding was associated with SSRI anti-anxiety effects and can be shaped by psychological expectancies[20]. CBT may affect dopamine D2 receptors [53] but, to our knowledge, no earlier PET-study has investigated concurrent changes in serotonin and dopamine transporters resulting from CBT or combined SSRI + CBT treatment.

The aim of this study was to evaluate changes in serotonin and dopamine transporter availability in SAD patients after 9 weeks of treatment with Internet-delivered CBT (ICBT) [54, 55] combined with an SSRI (escitalopram) or pill-placebo under double-blind conditions, and if such changes are associated with symptom improvement. In a PET subsample of a previously reported RCT [4], we measured SERT and DAT binding with the two highly selective radioligands [^11^C]DASB and [^11^C]PE2I. We expected marked SERT occupancy (lowered binding potential) specifically with SSRI-treatment whereas we did not have directed hypotheses regarding DAT changes. We also examined the relationships between transporter co-expression at baseline and symptom severity as well as symptom remission with treatment.

## Patients and methods

### Participants and design

The study was a double-blind clinical trial with a treatment duration of 9 weeks (trial registration: ISRCTN24929928), conducted between September 2011 and September 2013, and details have been described previously [4]. Briefly, participants were recruited through media advertisements and screened for SAD using online versions of the Social Phobia Screening Questionnaire (SPSQ) [56] and the Montgomery-Åsberg Depression Rating Scale–self rated version (MADRS-S) [57]. The Mini International Neuropsychiatric Interview (MINI) [58], and the SAD section of the Structured Clinical Interview for DSM-IV (SCID-I) [59] were thereafter administered via telephone and subjects deemed to fulfill the criteria for SAD went through a medical examination.

Exclusion criteria were previous PET-scan, treatment of any psychiatric condition during the last three months, ongoing serious somatic or psychiatric disorder, drug or substance abuse or dependency, menopause, pregnancy and MRI contraindications. All subjects provided written informed consent. The study was approved by the regional ethics committee, the Radiation Safety Committee and the Medical Products Agency in Sweden.

After screening, 48 subjects were enrolled and randomized to treatment with SSRI + ICBT or placebo+ICBT [4]. SAD was the primary diagnosis for all participants. Randomization, matched for age and sex was performed by Apoteket Production and Laboratories (APL), Stockholm, Sweden. All subjects underwent functional and structural MRI and 26 of these participants were again matched on age and sex and underwent additional PET assessments. The PET pairs were drawn from the whole sample, although no random sampling was applied. The Uppsala University Hospital Pharmacy kept all randomization codes secret until unblinding was due. One participant opted out of the post-treatment PET assessment, and one participant dropped out of the study before scanning procedures, leaving 24 participants (12 women) with complete PET data. There were 6 women and 6 men in each group and mean age±SD was 37.2 ± 11.32 and 31.25 ± 8.11 years for the SSRI + ICBT and the placebo + ICBT groups respectively. Sex and age distributions were not significantly different between the groups (*P*s > 0.156).

### Treatment

During the 9-week treatment period, the SSRI group was prescribed with a 20 mg daily dose of escitalopram, (H.Lundbeck AB, Helsingborg, Sweden) starting with 10 mg daily dose during the first week. SSRI and placebo oral suspension capsules were identical, and prepared by APL, Stockholm, Sweden. Compliance to escitalopram treatment was assessed by analyzing blood metabolites of escitalopram at the time for the last PET-scan. SSRI and placebo treatments were supervised by an experienced psychiatrist (K.W).

In tandem with the first SSRI or placebo therapy, ICBT was started as well. The ICBT program has been found to be effective in several RCTs [55, 60, 61], and found to be as effective as face-to-face CBT [54, 62]. The program is partly based on the Clark and Wells model of SAD [63] and includes 9 weekly modules: Introductory reading about SAD and CBT (module 1), the cognitive model for SAD and cognitive restructuring (modules 2–4), exercises for exposure (modules 5–7), social skills and relapse prevention (modules 8–9). The treatment was guided by trained therapists who gave the participants weekly homework, feedback on assignments, and introduced the next week’s module. All homework and completion of modules were registered to assess participants’ compliance to the treatment [4].

### Behavioral measures

Severity of social anxiety symptoms was measured using the clinician-administered Liebowitz Social Anxiety Scale [64] before and after treatment. Treatment response was assessed using the Clinical Global Impression Improvement (CGI-I), scores of 1 or 2 denoting responders and ≥3 non-responders. Both instruments were administered by the same experienced psychiatrist.

### Imaging procedure

#### PET-assessments

Participants were instructed to fast for 3 h before the scanning session and to abstain from nicotine, alcohol and caffeine 12 h before. Image acquisition was performed using a Siemens ECAT EXACT HR + 32-ring high-resolution scanner with 63 contiguous 2.46 mm slices. Participants were placed in the PET scanner with their heads lightly fixated and a transmission scan was performed using 3 retractable Germanium 68 rotating line sources for 10 min before the tracer was delivered with a rapid bolus injection through a venous catheter inserted in the arm. Dynamic PET image acquisition was initiated at the time of bolus injection. For both [^11^C]DASB and [^11^C]PE2I, the same procedure was used during pre- and posttreatment.

*[*^*11*^*C]PE2I image acquisition:* Twenty-two [^11^C]PE2I images, probing DAT availability, were collected during 80 min (4 × 60s, 2 × 120s, 4 × 180s, 12 × 300s). Mean ± SD injected activity was 332.38 ± 16.93 MBq at pretreatment and 319.33 ± 29.01 MBq at posttreatment.

*[*^*11*^*C]DASB image acquisition:* After a waiting period of 45–60 min to allow sufficient radioligand decay (less than 1% [^11^C]PE2I activity left at [^11^C]DASB injection), 22 [^11^C]DASB images, probing SERT availability, were collected during 60 min (1 × 60s, 4 × 30s, 3 × 60s, 4 × 120s, 2 × 180s, 8 × 300s). Mean±SD injected activity at pre-treatment was 329.50 ± 26.90 and at post- treatment, 318.75 ± 37.62 MBq.

#### MRI-assessments

Anatomical MRI was performed to allow co-registration of PET images to anatomical T1-weighted images. The T1 images (echo time (TE) = 15 ms; repetition time (TR) = 5700 ms; inversion time = 400 ms; field of view = 230 × 230 mm^2^; voxel size = 0.8 × 1.0 × 2.0 mm^3^; 60 contiguous axial slices) were acquired with a Philips Achieva 3.0 T whole body MR scanner (Philips Medical Systems, Best, The Netherlands) using an 8-channel head coil.

#### Preprocessing

Parametric [^11^C]PE2I binding potential (BP_ND_) images were generated using receptor parametric mapping [65] which is based on a simplified reference region compartmental model and [^11^C]DASB BP_ND_ images were calculated using the reference Logan [66] method. When using reference Logan, BP_ND_ is derived by subtracting 1 from the distribution volume ratio. Cerebellum gray matter was used as a reference region for both [^11^C]DASB and [^11^C]PE2I due to its negligible levels of SERT and DAT. The cerebellum was defined in a user-independent fashion using the PVElab [67] software on each participant’s T1-weighted image, which was co-registered to the PET images.

Further preprocessing steps were performed in Statistical Parametric Mapping 8 (SPM8; (Wellcome Department of Cognitive Neurology, University College London, www.fil.ion.ucl.ac.uk) implemented in MATLAB 2018a (Mathworks Inc., Natick, MA, USA). Each participant’s BP_ND_ images were co-registered to the anatomical T1-weighted image, which was then segmented and normalized to MNI standard space. Transformation parameters from segmentation were then applied to the BP_ND_ images yielding parametric images with 2 mm isotropic voxels in MNI space. Lastly, images were smoothed with a 12 mm Gaussian kernel.

### Statistical analysis

To examine treatment effects on SERTs and DATs, pre to post treatment diff-images were prepared. SSRI occupancy images (100 × ([PRE − POST] / PRE)) of [^11^C]DASB BP_ND_ and percent change images (100 × ([POST − PRE]/PRE)) of [^11^C]PE2I BP_ND_ were calculated for the SSRI + ICBT treatment arm. Occupancy is a measure of the proportion of the transporters available pre-treatment that are occupied by SSRI at post-treatment. Hence, occupancy > 0% is reflected by a decrease in binding potential after treatment, whereas percent change > 0% signifies an increase in binding potential. Since there is no SERT occupancy of SSRIs in the placebo+ICBT treatment arm, percent change images were calculated for both [^11^C]DASB BP_ND_ and [^11^C]PE2I BP_ND_. The LSAS post-scores were subtracted from the pre measurement scores with higher positive scores reflecting a larger symptom improvement.

A priori regions of interest (ROIs) were selected based on earlier neuroimaging research in SAD and tracer binding [13, 68, 69]. [^11^C]PE2I binding is specific to regions rich in dopamine transporters, such as the dorsal (putamen, caudate nucleus) and ventral striatum (nucleus accumbens (NAcc), but adequate levels can also be found in the amygdala, hippocampus, thalamus and pallidum. BP_ND_ distributions were tested for heterogeneity and normality and were deemed adequate for parametric analyses. [^11^C]DASB BP_ND_ analyses were performed in the same ROIs but also extended to the insular cortex, anterior cingulate cortex (ACC) and the raphe nuclei. Anatomical regions were defined by masks available in the Automated Anatomical Labeling (AAL) library found in the Wake Forest University Pickatlas [70]. The raphe nuclei were defined using PVElab, and NAcc with the Hammersmith atlas [71].

#### Within group effects

Effects of treatment on SERT and DAT BP_ND_ were evaluated with one-sample *t*-tests using the occupancy and percent change diff-images for the two tracers separately. Associations between changes in transporter binding and symptom reduction were evaluated in SPM 8 using multiple regressions with age and sex as covariates. Family wise error (FWE) correction was used within each ROI and the statistical threshold was set at *P*_FWE_ < 0.05. Co-expression of SERT and DAT was analyzed in Matlab 2018a, (Mathworks Inc, Natick, MA, USA) using voxel-wise partial Pearson’s correlations (age and sex as controlling variables) with the statistical threshold set at *P* < 0.05. To examine if changes (Δ) in SERT and DAT availability were associated with altered symptom severity, multiple regressions were performed with ΔSERT, ΔDAT, their interaction term, age and sex, as regressors. A similar regression using initial values of SERT and DAT BP_ND_ and their interaction term as predictors, and the change in LSAS as outcome, examined if initial SERT-DAT balance predicted treatment outcome.

#### Between group effects

To examine treatment group differences in changes in DAT binding, a two-sample *t*-test was performed in SPM8 with age and sex as covariates and the statistical threshold set to *P*_FWE_ < 0.05. Additionally, a multiple regression was used to compare the relation between the percentage change in DAT BP_ND_ and symptom reduction between groups. Note that the high affinity of escitalopram to the SERT precluded the possibility of group comparisons of changes in SERT and SERT × DAT interactions.

## Results

### Treatment outcome

Initial LSAS scores and depression comorbidity data are found in Table 1. No initial difference in social anxiety symptoms was found between the two groups (*t*_(22)_ = 0.55, *P* = 0.59). Repeated measures ANOVA revealed statistically significant symptom improvement (LSAS scores) in both groups from pre to post-treatment (*F*_(1, 22)_ = 43.12, *P* < 0.001, Cohen’s *d* = 1.32). Follow up *t*-tests verified significant symptom improvement in the SSRI + ICBT (*t*(_11_) = 5.16, *P* < 0.001) as well as in the placebo+ICBT group (*t*(_11_) = 4.07, *P* = .002). No effect of group on symptom improvement (*F*(_1,22)_ = 1.51, *P* = 0.232) or group × time effect (*F*_(1,1)_ = 1.83, *P* = 0.186) was detected. According to CGI-I assessments, there were 10 responders (83%) in the SSRI + ICBT group and 5 (42%) in the placebo+ICBT group (Fisher’s exact test: *P* = 0.089), congruent with the generally better outcome for SSRI + ICBT reported in the full treatment sample [4].

**Table 1:** Mean (SD) scores of social anxiety (LSAS) and depression (MADRS-S) pre and posttreatment including MADRS-S depression category at pretreatment.

	Pre	Post	Diff	
LSAS				
SSRI+CBT	71.50 (27.17)	34.91 (20.91)	−36.59	
Placebo+CBT	77.33 (24.33)	53.33 (32.85)	−24.00	
MADRS-S				
SSRI+CBT	13.17 (9.27)	3.33 (2.71)	−9.84	
Placebo+CBT	14.75 (10.36)	6.33 (5.63)	−8.42	
MADRS-S category (pre)	No	Mild	Moderate	Severe^a^
	8	2	1	1
	6	4	1	1

^a^Not deemed severe after clinical interview.

### Serotonin transporter binding

**Fig. 1:**
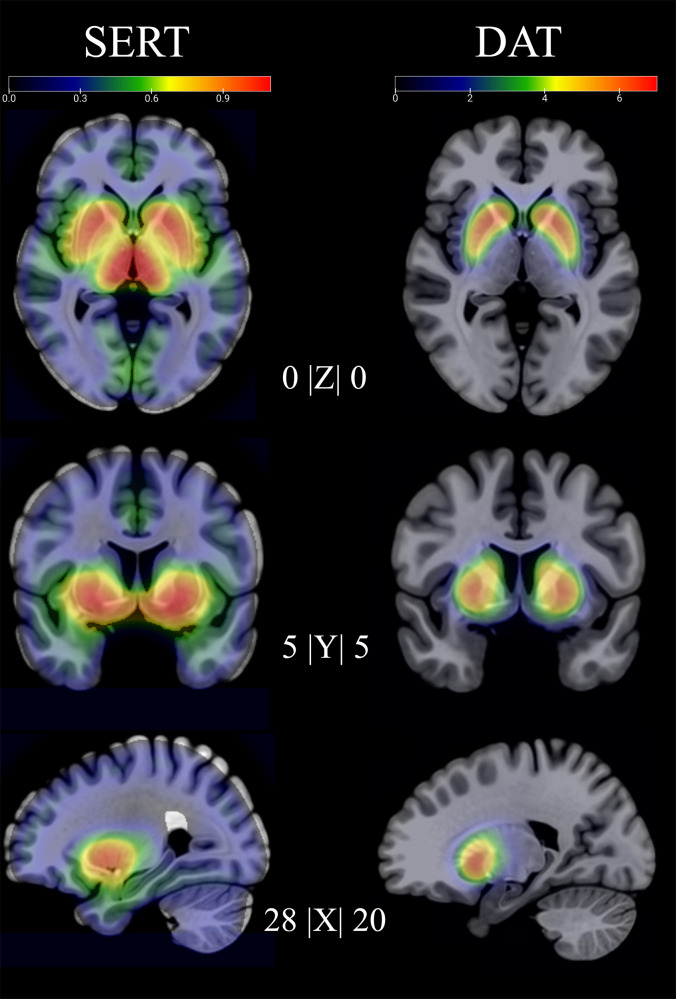
Whole sample pre-treatment SERT and DAT binding. Left panel shows serotonin transporter non-displaceable binding potential pre treatment and the rightpanel shows non-displaceable binding potential for the dopamine transporter.

Groups did not differ in initial SERT binding (see Fig. 1 for whole sample SERT and DAT BP_ND_). Symptom severity, measured with LSAS, showed negative associations with whole sample SERT BP_ND_ in the left dorsal ACC as previously reported [13]. Injected activity for both tracers can be found in Supplementary Table 1.

**Fig. 2:**
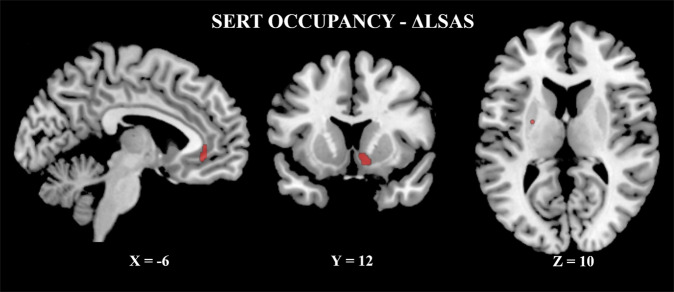
PET results from the SSRI + ICBT group. Regions where significant positive associations between SERT occupancy and symptom improvement were detected, i.e., the left anterior cingulate, right nucleus accumbens and left putamen.

Regarding SSRI + ICBT treatment effects, the mean occupancy of the SERT across the a priori selected ROIs was >80% (Table 2, Supplementary Table 2) and there were significant positive relations between SERT occupancy and symptom improvement in the right NAcc, left putamen and left ACC in this group (Table 3, Fig. 2). The mean (±SD) concentrations (nMol/l) of blood serum escitalopram and desmethylescitalopram after treatment were 78.3 (±42.7) and 35.0 (±14.4) respectively in the SSRI + ICBT group. Concentrations were 0 for the same measures after placebo+ICBT. Neither of these measures were significantly correlated with symptom improvement (*P* > 0.27) or SERT occupancy (*P* > 0.11).

**Table 2:** Serotonin (SERT) and dopamine (DAT) transporter binding potential (BP_ND_) changes in patients with social anxiety disorder (SAD) after treatment with SSRI + ICBT or placebo + ICBT.

	x	y	z	*Z*	*P*_*FWE*_	Cluster volume^1^
Serotonin transporter (SERT)						
SSRI + ICBT						
Occupancy						
All regions				*Inf*		
Placebo + ICBT						
Increase BP_ND_						
Raphe	−4	−30	−28	3.31	0.009	632
Dopamine (DAT)						
SSRI + ICBT						
Increase						
L Amygdala	−28	4	−18	3.41	0.008	752
R Amygdala	18	0	−18	3.59	0.005	1432
L Hippocampus	−36	−18	−14	3.92	0.006	376
R Hippocampus	30	−28	−6	3.36	0.035	24
L NAcc	−12	12	−12	3.27	0.005	264
R NAcc	12	12	−12	3.09	0.007	168
L Putamen	−24	12	−10	3.44	0.026	328
Placebo + ICBT						
Increase						
L Amygdala	−24	4	−18	3.58	0.006	968
R Amygdala	20	6	−18	4.04	0.002	1440
L Hippocampus	−20	−20	−14	3.36	0.040	16
R Hippocampus	28	−16	−22	4.43	0.001	568
L NAcc	−12	10	−14	3.71	0.001	224
R NAcc	12	12	−12	3.04	0.009	56
L Putamen	−16	16	−10	3.28	0.046	16
R Putamen	30	12	−8	3.86	0.009	504

*MNI* Montreal Neurological Institute, *L* left, *R* right, *NAcc* Nucleus Accumbens.

^a^Cluster volume in mm^3^.

**Table 3:** Relations between treatment-induced changes in symptom severity, as measured by the Liebowitz social anxiety scale (LSAS), and corresponding changes in serotonin transporter (SERT) occupancy and dopamine (DAT) transporter binding potential (BP_ND_).

	x	y	z	Z	*P*_*FWE*_	Cluster volume^a^
Serotonin transporter						
SSRI^b^ + ICBT^c^						
Positive						
R NAcc	6	10	−12	3.62	0.002	272
R Caudate (NAcc)	6	12	−10	3.51	0.034	48
L Putamen	−26	−4	10	3.42	0.042	8
L ACC	−8	40	−6	3.57	0.007	8
Placebo + ICBT^c^						
–						
Dopamine transporter						
SSRI + ICBT^d^						
Negative						
L Amygdala	−28	−4	−24	2.96	0.035	8
Placebo + ICBT^d^						
Positive						
L NAcc	−6	8	−8	2.97	0.015	336
SSRI + ICBT < Placebo + ICBT						
L NAcc	−4	10	−6	3.55	<0.001	512
R NAcc	4	8	−8	2.71	0.018	56
L Thalamus	−14	−26	2	3.53	0.016	376

*MNI* Montreal Neurological Institute, *L* left, *R* right, *NAcc* Nucleus Accumbens, *ACC* Anterior Cingulate Cortex.

^a^Cluster volume in mm^3^.

^b^Selective Serotonin Reuptake Inhibitor, escitalopram.

^c^High SSRI occupancy reflects decreased BP_ND_.

^d^Percent change of BP_ND_.

In the placebo+ICBT arm, there was a significant increase in SERT binding potential from pre- to posttreatment in the raphe nuclei (Table 2) and, when applying a more lenient statistical threshold (*P*_FWE_ < 0.10), also in the right amygdala (*P*_FWE_ = 0.068, MNI: 20 4 –16, *Z* = 2.63), right putamen (*P*_FWE_ = 0.056, MNI: 20 18 –10, *Z* = 3.16) and right NAcc (*P*_FWE_ = 0.061, MNI: 12 12 –12, *Z* = 2.18). No significant associations between change in SERT binding and symptom improvement were detected in the placebo+ICBT group.

### Dopamine transporter binding

**Fig. 3:**
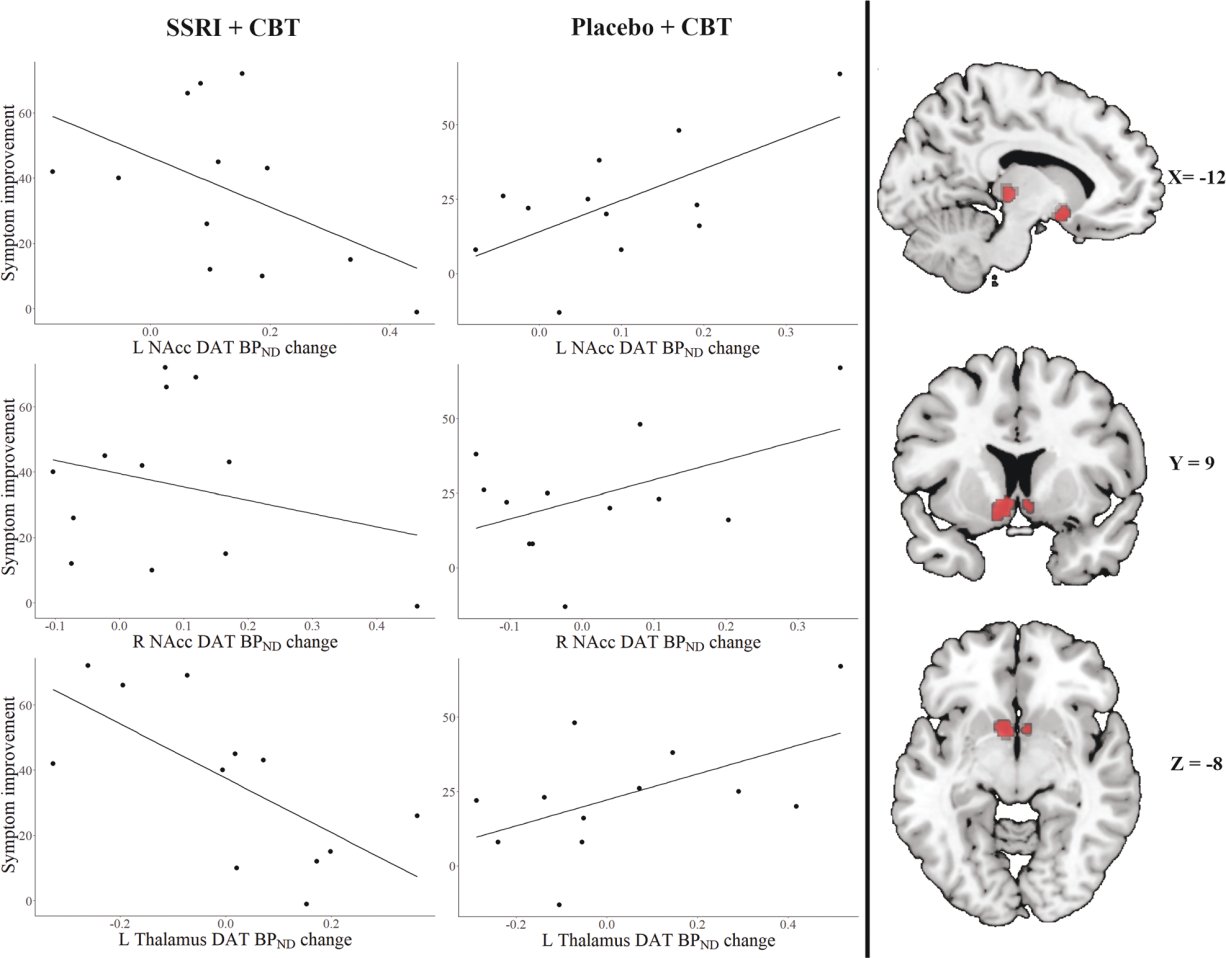
Scatterplots of significant group differences in associations between symptom improvement, as measured with the Liebowitz Social Anxiety Scale (LSAS), and the percentage change in DAT BPND in the left nucleus accumbens (L NAcc), right NAcc (R NAcc) and left thalamus. Clusters of significant voxels (*P*_FWE_ < 0.05) shown overlaid on a standard anatomical brain template.

Baseline DAT BP_ND_ did not differ between groups and was not significantly related to symptom severity. Both groups showed increases in DAT availability after treatment in the bilateral amygdala, hippocampus, NAcc, and putamen (Table 2). However, groups differed significantly in their association between the pre-post change in DAT BP_ND_ and symptom improvement (LSAS) (Table 3, Fig. 3). In the SSRI + ICBT group, increased DAT BP_ND_ in the left amygdala (Table 3) and less robustly also in the left NAcc (*P*_FWE_ = 0.087, MNI: −6 12 −8, *Z* = 2.12), was related to lesser symptom improvement. Conversely, in the placebo+ICBT group, increased DAT binding in the left NAcc was associated with larger improvement (Table 3).

### Serotonin-dopamine transporter co-expression

At baseline, voxel-wise regressions of binding potentials for the whole sample revealed significant SERT-DAT co-expression (positive beta values) in all ROIs, which remained for most regions at post treatment (Supplementary Table 3). Higher baseline co-expression in the left putamen and left thalamus was associated with more severe social anxiety (Supplementary Table 4). In SSRI + ICBT subjects, higher pre-treatment SERT-DAT co-expression in the right NAcc, left putamen, right pallidum and right thalamus, significantly predicted symptom reduction with treatment (Supplementary Table 5). In placebo + ICBT subjects, the same pattern was found in the thalamus, whereas in the right amygdala, bilateral hippocampus, left putamen and right pallidum initially lower SERT-DAT co-expression was significantly associated with larger symptom reduction following treatment. Neither SERT nor DAT binding at baseline was predictive of treatment outcome by themselves. See also supplementary material.

## Discussion

By use of PET and two selective radioligands, we examined parallel changes in serotonin and dopamine transporters resulting from 9 weeks of combined pharmacologic (SSRI) and psychological (ICBT) treatment for social anxiety under double-blind randomized conditions. Both SSRI + ICBT and placebo + ICBT resulted in significant improvement on the main social anxiety outcome (LSAS), with a trend towards higher number of responders in the SSRI arm. Since the clinical measures of the full cohort has already been evaluated [4], the aim of the current study was not to verify differential treatment efficacy, but to evaluate if monoaminergic transporter mechanisms underlying symptom improvement differ between the two treatment modalities. Both treatment combinations yielded similar pre-to-post increases in DAT availability in limbic and striatal regions but associations with symptom reduction differed in direction across treatment groups. Baseline SERT-DAT co-expression was high, and showed positive relations with initial symptom severity. Co-expression also predicted treatment outcome, albeit again in different directions in the two groups.

As expected, only the SSRI + ICBT combination yielded a SERT occupancy rate consistent with SSRI efficacy. The mean level of all investigated ROIs was >80%, indicating good compliance with SSRI medication, as also verified by analyses of serum metabolites. It has been suggested that an occupancy rate of 76–85% is sufficient to yield a therapeutic effect [17, 18]. Moreover, in the NAcc, putamen and ACC, SERT occupancy was significantly associated with symptom improvement which has not been reported before in SAD [19, 20], but has been infrequently observed in other disorders like geriatric depression [25]. With the current design, it cannot be excluded that ICBT moderated this effect.

Studies of depression have suggested that SERT binding increases after CBT [72, 73]. Consistently, in the placebo+ICBT arm, we observed increased (pre-post) SERT binding in the raphe nuclei, although this effect was not related to clinical improvement. The raphe is regarded as an important target for SSRIs due to its high concentration of serotonergic neurons, and PET studies of SAD have demonstrated lower serotonin 1 A binding [14] and increased serotonin synthesis [13] in this region before treatment. The current results indicate that raphe serotonergic activity could also be modulated by ICBT, in line with findings of reduced serotonin 1B receptor binding in raphe after ICBT for major depressive disorder [74]. Increased SERT binding, suggesting faster serotonin clearance, is interesting in the context of reduced serotonin synthesis reported after anxiolytic treatment [68]. However, ICBT effects on raphe SERTs did not occur concomitantly with altered serotonin transport in other regions.

Regarding dopamine transport, both groups exhibited a general increase in DAT binding with treatment. In SAD, similar effects of escitalopram have previously been reported in a SPECT study, where DAT increases were limited to the left dorsal striatum and did not correlate with symptom improvement [48]. Additionally, in a recent PET study from our group on SSRI response expectancies [20], DAT BP_ND_ increases were found in the hippocampus and pallidum, but only in the treatment group with lowered expectancies of improvement induced by verbal instructions. Since similar DAT increases were observed in both groups in the current study, it is possible that these changes were mainly driven by ICBT, i.e., dopaminergic changes might be more pronounced with ICBT than SSRI-treatment.

Despite common DAT increases, the two treatment groups showed inverse associations between DAT BP_ND_ change and treatment outcome. With SSRI + ICBT, symptom improvement was negatively associated with DAT change in the amygdala. Further, in the ventral striatum and thalamus, symptom improvement was associated with smaller DAT increases in the SSRI + ICBT arm relative to placebo+ICBT where positive associations were noted. Similarly, we previously observed that pre-post reductions in DAT improved symptoms in SAD patients treated openly with escitalopram [20]. The current data, however, suggests a different role of dopamine in ICBT. Stronger association between increased DAT BP_ND_ in NAcc and symptom reduction with placebo+ICBT could possibly reflect that ICBT has a greater influence on the ventral striatum, known to be important for approach-avoidance conflict resolution, reward processing and plays a major role in placebo responses [75–77]. The noted association between ICBT outcome and the overall DAT increases suggest that further study of appetitive/approach elements of CBT and their associations with dopamine function is warranted. Speculatively, SSRIs may act more by modulating amygdala threat signaling. For example, SSRI + ICBT yielded stronger attenuation of amygdala BOLD reactivity to emotional faces than placebo+ICBT in the larger cohort [4]. Very few studies have examined dopaminergic changes in SAD, non-confounded by pharmacological treatment, but Cervenka and coworkers [53] found increased D2 receptor binding potential in limbic and pre-frontal areas with ICBT. Since changes both in D2 and DAT parameters can be linked to treatment outcome in SAD, and since D2 autoreceptors regulate dopamine synthesis and DAT expression [78], further research on both dopamine sub-systems is warranted. As we mentioned previously in a study of a different cohort [20], our PET data may indicate dopaminergic dysfunction in SAD similar to at least some subgroups of treatment resistant depression for which dopamine agonists could be effective [79]. However, dopaminergic medications have not stood out as particularly effective on their own, although this topic is understudied [80, 81].

In all evaluated brain regions, there was significant positive co-expression of SERT and DATs at baseline which exhibited a positive relation with symptom severity. We have reported a similar relationship in a different cohort, i.e., a significantly higher correlation coefficient between SERT and DAT BP_ND_ in SAD patients relative to healthy controls [15]. Thus, upregulated monoamine co-expression could be involved in the pathophysiology of SAD. Differences were also noted regarding prediction of treatment outcome. In the SSRI + ICBT group, high initial SERT-DAT co-expression in striatal-thalamic areas predicted better treatment outcome, which was also found for the thalamus in the placebo+ICBT group, whereas for other brain regions, high initial SERT-DAT co-expression was generally disadvantageous for treatment success with placebo+ICBT. Similarly, in the larger cohort, we previously demonstrated that initial neural activations of the dACC in response to emotional faces, predicted outcome in different directions in the two treatment modalities [82].

The multi-tracer PET methodology enabling analysis of transporter co-expression, the double-blind RCT design, and inclusion of a non-pharmacologic treatment group are major strengths of our study, but there are also limitations to consider. First, an additional control group to the ICBT condition, e.g., a waiting-list, no-treatment, or placebo-only control, would have been helpful to capture the complete contribution of ICBT. Second, voxel-based analyses are likely more spatially sensitive than regional mean approaches but might also be more susceptible to noise due to the smaller number of activity counts detected within the limited volume and due to smoothing of parametric images especially in smaller ROIs. Also, the complex dynamics between serotonin and dopamine signaling cannot be uncovered by PET data on transporters only and the longevity of the transporter changes needs further evaluation. Another limitation is that analyses were not adjusted for menstrual cycle phase. Moreover, although PET is a more sensitive and precise imaging technique than SPECT, the restricted sample size warrants some caution, especially regarding the SERT×DAT interactions linked to symptom reduction (see supplementary material), because the number of regressors were large in relation to sample size.

In conclusion, the current study replicates and extends several of our previous PET findings in SAD [15, 20], mainly that SAD patients before treatment exhibit strong positive SERT-DAT associations related to symptom severity, that clinical doses of escitalopram result in high (>80%) SERT occupancy, here associated with clinical improvement, and that reductions or lesser increases of DAT availability are associated with better outcome in SSRI-treated patients. Results further suggest that monoamine transporter co-expression has an impact on symptom remission with treatment and that pharmacologic and psychosocial treatments modulate the transporter proteins in disparate ways.

## Supplementary information

Supplementary Material

CONSORT Flow chart
