# Supplementary material for: Serotonin and dopamine transporter availability in social anxiety disorder after combined treatment with escitalopram and cognitive-behavioral therapy
Source: Transl Psychiatry. 2022 Oct 7;12:436. doi: 10.1038/s41398-022-02187-3 (PMC9537299; doi:10.1038/s41398-022-02187-3)
Supplement: Supplementary file 1 — Supplementary Material [file 41398_2022_2187_MOESM1_ESM.docx]

**Supplementary material:**

**Serotonin and dopamine transporter availability in social anxiety disorder after combined treatment with escitalopram and cognitive-behavioral therapy**

**Supplementary Table 1.** Mean(Minimum) Injected activity in MBq for both tracers and groups before and after treatment.

|  | **PE2I** | | **DASB** | |
| --- | --- | --- | --- | --- |
|  | **PRE** | **POST** | **PRE** | **POST** |
| **SSRI+CBT** | 325.5(212) | 316.5(259) | 322.6(248) | 329.8(300) |
| **PLACEBO+CBT** | 331.3(295) | 322.6(240) | 337.6(309) | 305.7(204) |

**Supplementary Table 2.** Escitalopram occupancy of the serotonin transporter for included ROIs and a total score for the full volume of all included regions of interest (ROIs).

| **ROI** | **Occupancy(%)** |
| --- | --- |
| Amygdala | 85.37 |
| Hippocampus | 88.68 |
| Insula | 77.22 |
| Putamen | 74.01 |
| Caudate | 95.29 |
| NAcc | 85.74 |
| Pallidum | 72.08 |
| Raphe | 85.17 |
| ACC | 81.11 |
| Thalamus | 76.09 |
| Volume total | 80.18 |

NAcc = Nucleus Accumbens; ACC = Anterior Cingulate Cortex

**Supplementary Table 3**. Significant co-expression of serotonin and dopamine transporters, as assessed with voxel-wise correlations of binding potentials (BP_ND_) using [^11^C]DASB and [^11^C]PE2I PET, in patients with social anxiety disorder. Statistical threshold set to *p*<.05

|  | x y z | | | *r*^1^ | Cluster volume^2^ |
| --- | --- | --- | --- | --- | --- |
| **Pre treatment** |  |  |  |  |  |
| **Whole sample** |  |  |  |  |  |
| R Amygdala | 24 | 2 | -24 | .536 | 3136 |
| R Hippocampus | 30 | -12 | -24 | .686 | 20992 |
| L Hippocampus | -18 | -36 | 6 | .598 | 768 |
| L NAcc | -4 | 10 | -6 | .543 | 1920 |
| R NAcc | 4 | 8 | -8 | .655 | 2304 |
| L Caudate | -12 | 8 | 16 | .872 | 30912 |
| R Caudate | 16 | 6 | 12 | .772 | 35648 |
| L Putamen | -20 | 4 | 14 | .794 | 18752 |
| R Putamen | 24 | 0 | 14 | .717 | 28800 |
| L Thalamus | -10 | -10 | 0 | .713 | 35904 |
| R Thalamus | 4 | -8 | 6 | .756 | 27136 |
| **Post treatment** |  |  |  |  |  |
| **SSRI+CBT** |  |  |  |  |  |
| L Amygdala | -28 | -6 | -12 | .829 | 3072 |
| L Caudate | 10 | 12 | 2 | .903 | 7296 |
| R Caudate | -10 | 16 | -2 | .778 | 2304 |
| L Putamen | -22 | 14 | 5 | .779 | 20200 |
| R Putamen | 30 | -14 | -4 | .828 | 13440 |
| L Thalamus | -10 | -26 | -2 | .924 | 13184 |
| R Thalamus | 8 | -22 | -2 | .791 | 3328 |
| **Placebo+CBT** |  |  |  |  |  |
| L NAcc | -4 | 6 | -6 | .753 | 2752 |
| R NAcc | 4 | 10 | -6 | .810 | 2368 |
| L Caudate | -12 | 8 | 16 | .834 | 11576 |
| R Caudate | 10 | 8 | 16 | .843 | 4928 |
| L Putamen | -20 | 6 | 10 | .742 | 1664 |
| R Thalamus | 8 | -26 | 0 | .802 | 6976 |
| **Change pre-post** |  |  |  |  |  |
| **SSRI+CBT** |  |  |  |  |  |
| L Thalamus | -10 | -28 | 2 | 0.714^3^ | 960 |
| **Placebo+CBT** |  |  |  |  |  |
| n.s |  |  |  |  |  |

MNI: Montreal Neurological Institute; NAcc = Nucleus Accumbens

^1^ Regional co-expression indexed by Pearson’s product-moment correlation coefficient, *r*.

in regional co-expression.

^2^ Cluster volume in mm^3^.

^3^ Difference in *r* between pre (.13) and post (.84)

**Supplementary Table 4.** Social anxiety symptom severity as a function of serotonin (SERT) and dopamine transporter (DAT) binding potential at baseline and after treatment with cognitive behavioral therapy (ICBT) combined with either the selective serotonin reuptake inhibitor (SSRI) escitalopram or pill placebo. Shown are clusters from voxel-wise linear regressions with social anxiety symptoms (LSAS) as outcome and SERT binding potential (BP_ND_), DAT BP_ND_ and their interaction term as regressors. Regressions of baseline BP_ND_ and symptom severity are also listed. Statistical threshold was set at P < .05

|  | x y z | | | SERT×DAT^1^ | *P* | Cluster volume^2^ |
| --- | --- | --- | --- | --- | --- | --- |
| **Baseline: Whole sample**  SERT×DAT^3^ |  |  |  |  |  |  |
| Left Putamen | -22 | 14 | 2 | 0.601 | .007 | 8064 |
| Left Thalamus | -18 | -26 | 0 | 0.781 | .008 | 12096 |
| **Treatment effects** Δ LSAS |  |  |  |  |  |  |
| SSRI+ICBT |  |  |  |  |  |  |
| [Δ SERT × Δ DAT] - Δ LSAS^4^ |  |  |  |  |  |  |
| Right NAcc | 10 | 6 | -12 | 0.509 | .024 | 512 |
| Left Thalamus | -4 | -22 | -2 | 0.913 | .041 | 1280 |
| Right Thalamus | 4 | -18 | 4 | 1.105 | .003 | 3456 |
| Placebo+ICBT |  |  |  |  |  |  |
| [Δ SERT × Δ DAT]- Δ LSAS^5^ |  |  |  |  |  |  |
| Left Putamen | -24 | 12 | 12 | -0.810 | .003 | 3328 |

MNI: Montreal Neurological Institute: L = left, R = right; NAcc = Nucleus Accumbens

^1^ *β* of LSAS~SERT+DAT+(SERT×DAT) + age + sex.

^2^ Cluster volume in mm^3^

^3^ Brain areas where high SERT-DAT BP_ND_ associations were related to symptom severity (LSAS) at baseline

^4^ Brain areas showing parallel pre-post changes in SERT (occupancy) and DAT (BP_ND_ percentage change) that were associated with symptom improvement (LSAS change scores)

^5^ Brain areas showing parallel pre-post changes in SERT and DAT (both BP_ND_ percentage change) that were associated with symptom improvement (LSAS change scores)

**Supplementary Table 5.** Predictions of symptom improvement based on initial transporter co-expression. Voxel-wise linear regression of social anxiety (LSAS) with SERT BP_ND_, DAT BP_ND_ and their interaction term as regressors are listed.

|  | x | y | z | **SERT×DAT^1^** | *P* | **Cluster volume^2^** |
| --- | --- | --- | --- | --- | --- | --- |
| **SSRI+CBT** |  |  |  |  |  |  |
| Pre SERT×DAT - Δ LSAS |  |  |  |  |  |  |
| R NAcc | 4 | 10 | -6 | 0.687 | .024 | 704 |
| L Putamen | -26 | 8 | -4 | 1.130 | .007 | 7808 |
| R Pallidum | 16 | 4 | -4 | -0.757 | .040 | 1152 |
| R Thalamus | 10 | -12 | 2 | 1.003 | .008 | 1920 |
| **Placebo+CBT** |  |  |  |  |  |  |
| Pre SERT×DAT - Δ LSAS |  |  |  |  |  |  |
| R Amygdala | 28 | -2 | -26 | -0.783 | .039 | 832 |
| L Hippocampus | -30 | -8 | -22 | -1.480 | .006 | 896 |
| R Hippocampus | 22 | -14 | -12 | -1.693 | .023 | 1152 |
| L Putamen | -30 | -14 | 8 | -1.070 | .006 | 576 |
| R Pallidum | 18 | -4 | -4 | -1.766 | .001 | 6400 |
| L Thalamus | -4 | -10 | -2 | 0.774 | .043 | 576 |
| R Thalamus | 8 | -20 | 0 | 1.027 | .049 | 768 |

MNI: Montreal Neurological Institute: L = left, R = right; NAcc = Nucleus Accumbens

^1^ *β* of LSAS~DASB+PE2I+(DASB×PE2I) + age + sex.

^2^ Cluster volume in mm^3^
