# Supplementary material for: Serotonin and dopamine transporter availability in social anxiety disorder after combined treatment with escitalopram and cognitive-behavioral therapy
Source: Transl Psychiatry. 2022 Oct 7;12:436. doi: 10.1038/s41398-022-02187-3 (PMC9537299; doi:10.1038/s41398-022-02187-3)
Supplement: Supplementary file 2 — CONSORT Flow chart [file 41398_2022_2187_MOESM2_ESM.doc]

**CONSORT 2010 Flow Diagram**

**Allocation**

**Analysis**

**Follow-Up**

**Enrollment**

Assessed for eligibility (n=153 )

Excluded (n=72 )

♦  Not meeting inclusion criteria (n=3 )

♦  Meting at least one exclusion criteria (n=41)

♦  Declined to participate (n=22 )

♦  Not able to reach for interview (n=6 )

PET data analysed (n=12 )

Lost to follow-up (opted out of post treatment PET) (n= 1 )

Allocated to escitalopram + ICBT (n=24 )

♦ Received allocated intervention (n=24 )

♦ Allocated to PET subgroup (n=13 )

Lost to follow-up (n=0 )

Allocated to placebo + ICBT (n=24 )

♦ Received allocated intervention (n=24 )

♦ Allocated to PET subgroup (n=12 )

PET data analysed (n= 12 )

Randomized (n= 48 )

Excluded (n=33 )

♦  Not meeting inclusion criteria (n=32 )

♦  Meting at least one exclusion criteria (n=14)

♦  Declined to participate (n=15 )

♦  Not able to reach for scheduling (n=2 )

Interviewed (n=81 )
